# Knowledge Assessment of Hospital Nursing Staff in Saudi Arabia Regarding *Clostridioides difficile* Infection: A Descriptive Cross-Sectional Study

**DOI:** 10.3390/nursrep15020074

**Published:** 2025-02-19

**Authors:** Abdulrhman Albougami, Saeed S. Banawas

**Affiliations:** 1 Department of Nursing Administration, College of Nursing, Majmaah University, Al-Majmaah 11952, Saudi Arabia; 2 Department of Medical Laboratories, College of Applied Medical Sciences, Majmaah University, Al-Majmaah 11952, Saudi Arabia; 3 Health and Basic Sciences Research Center, Majmaah University, Al-Majmaah 11952, Saudi Arabia

**Keywords:** *Clostridioides difficile*, cross-sectional survey, infection diseases, medical laboratory, nurses, spore-forming bacillus

## Abstract

*Clostridioides difficile* (*C. difficile*), a gram-positive, spore-forming bacillus, has emerged as a leading cause of healthcare-associated infections, significantly contributing to infectious diarrhea and increasing healthcare costs. This descriptive, cross-sectional study was conducted among Saudi Arabian nursing staff from July to December 2023 to assess their knowledge and practices related to the diagnosis and management of *C. difficile* infection (CDI). Data were collected using a modified questionnaire. Overall, 358 nurses were surveyed, and 66% reported knowledge of *C. difficile* procedures. However, only 30.4% of the respondents correctly classified *C. difficile* as an anaerobic bacillus, while 42.2% were aware of the organism’s common occurrence in healthy adult volunteers. Additionally, 55.6% of respondents were aware of risk factors and 48.9% could name typical medicines that might cause illness. Only 24.0% acknowledged the cytotoxin test as the gold standard for detection, 26.8% identified hand washing with water and soap as an effective method to prevent the transmission of CDI, and 36.3% identified oral metronidazole as the first-line treatment for CDI. In summary, this study revealed a significant lack of awareness among nurses in Saudi Arabia regarding various aspects of CDI, emphasizing the need for improved education and training to address the knowledge gaps and quality of patient care.

## 1. Introduction

*Clostridioides difficile (C. difficile)*, a gram-positive, anaerobic, spore-forming bacillus, is recognized as an opportunistic pathogen found in the intestinal tracts of humans and animals, as well as in the environment [1,2,3,4,5,6,7]. Recent studies have highlighted its emergence as a leading cause of healthcare-associated infections (HAIs) [1,2,3]. Furthermore, *C. difficile* has been reported as the primary cause of infectious diarrhea in developed countries [4,6]. Pooled estimates from a systematic review suggest that CDI imposes a large burden on the United States (US) healthcare system [7]. In Saudi Arabia, CDI is reported to affect 6.8–9.1% of the population [8,9].

Antibiotic exposure is closely linked to CDI frequency by altering the gut microbiota and weakening colonization resistance [10,11,12]. Antibiotics disrupt the synthesis of secondary bile acids, promoting *C. difficile* colonization and active infection [13]. This disruption weakens the host’s ability to convert primary bile acids into secondary bile acids, facilitating *C. difficile* spore germination and infection, with taurocholate playing a key role [13]. There is a substantial correlation between antibiotic use and CDI [13,14]. Antibiotics such as penicillin, cephalosporins, clindamycin, and fluoroquinolones are associated with the highest risk of CDI [15,16,17]. Studies suggest that elderly individuals (i.e., those ≥65 years old) are ten times more susceptible to CDI than younger patients [18]. Other risk factors include hospitalization, gastrointestinal surgery, immunosuppressive conditions, organ transplantation, chemotherapy, inflammatory bowel disease, chronic renal disease, environmental contaminant exposure, contact with known carriers, and a history of CDI [17,19]. The role of stomach acid suppression in CDI is debated, but it may increase the risk of infection by altering gut microbiota [20]. Studies have shown that *C. difficile* colonization rates increase from 2% to 20% with longer hospital stays, prolonged antibiotic use, close contact with infected individuals, and nursing home residence [21,22,23]. *C. difficile* spores, highly resistant and commonly found in healthcare environments, underscore the need for strict hand hygiene and infection control protocols [24]. Effective measures include using disposable gloves and gowns, washing hands with soap and water, isolating infected patients, daily cleaning of high-touch surfaces, and thorough room decontamination upon patient discharge [24]. The success of these measures relies on the active participation of healthcare workers.

Nurses play an important role in preventing and managing CDI through the early recognition of symptoms, implementing infection control measures, educating patients, adhering to clinical guidelines, and monitoring infection rates [24,25]. Evidence from the published studies highlight the importance of nurses’ knowledge and adherence to the guidelines in managing CDI [26,27,28,29]. For instance, a cross-sectional study found that nurses with higher education levels and specific training on CDI had better knowledge and practices related to infection control [29]. Another study emphasized the role of nurses in critical care settings, where rapid diagnosis and treatment are crucial to reduce morbidity and mortality associated with CDI [27]. A descriptive, cross-sectional study found that only 27.9% of healthcare providers (physicians and clinical pharmacists) in Saudi Arabia had adequate knowledge of CDI diagnosis and management [30]. However, no studies have investigated the awareness levels of CDI among nurses in Saudi Arabia. Therefore, this study aimed to assess the awareness of CDI among nurses in Saudi Arabia. Specifically, this study aimed to identify knowledge gaps and improve future infection control training programs for nurses in Saudi Arabia.

## 2. Materials and Methods

### 2.1. Study Design and Setting

This descriptive, cross-sectional study was conducted among nursing staff from three tertiary university hospitals in Riyadh, Saudi Arabia, from July to December 2023.

### 2.2. Study Population

Nurses with a diploma, bachelor’s, master’s, or a doctoral degree and who expressed willingness to participate were included in this study. A total of 403 nurses were selected from the three university hospitals using a random sampling method to eliminate selection bias and ensure a balanced representation of various profile variables such as age, sex, experience, and education. In brief, a list of eligible nurses was documented for each hospital, and samples were randomly chosen based on the hospital’s size and required sample size. If a selected nurse was unavailable or uncooperative, they were randomly replaced with another eligible nurse from the same hospital. Nurses were informed about the study’s purpose, significance, methods, and potential benefits, and they received guidance on completing the questionnaire. Participants were encouraged to ask questions for clarity. Nurses who provided their written informed consent to participate received a link to the questionnaire.

### 2.3. Survey Questionnaire

A previously reported 17-item questionnaire was modified and used in this study [31]. The modified semi-structured questionnaire included 19 close-ended questions aimed at gathering demographic information about nurses and assessing their awareness and knowledge regarding CDI. The first three questions gathered demographic data on nurses, questions 4–6 focused on awareness of CDI-specific trust policy, field of expertise, and the timing of information. Subsequently, questions 7–10 covered microbiology and CDI-caused illnesses; questions 11–14 delved into risk factors, causes, prevention measures, and target population; questions 15–19 addressed CDI detection, prevention, and management (Supplementary File S1). The nurses’ perceptions of individual questions were recorded using a three-point scale. The questionnaire, designed for scientific purposes, was in English and included relevant instructions to help the respondents provide their answers. The questionnaire was administered online to all nurses and required approximately 10–15 min to complete, while ensuring their privacy and data security.

### 2.4. Outcome Measures

A survey questionnaire was used to collect data on demographic characteristics (age, sex, and education level). Additionally, information on the knowledge, attitudes, and practices related to CDI among nurses was collected.

### 2.5. Ethical Consideration

The study protocol and questionnaire received exemption approval from the Institutional Review Board of Majmaah University (Approval No. MUREC- Jan.l7 /COM-2023/3-4). Participants were assured of anonymity, safety, and confidentiality as well as their right to withdraw from this study at any time. All participants provided written informed consent after being informed about this study’s objectives and their privacy rights.

### 2.6. Statistical Analysis

Descriptive statistics, which included frequency distribution and percentages were compiled to summarize the demographic data and questionnaire results. Association between demographic characteristics and the participant responses was assessed using χ^2^ test. Data analyses were conducted using the Statistical Package for the Social Sciences (SPSS) version 27.0 (IBM Corporation, New York, NY, USA).

## 3. Results

Of the 403 nurses, 358 agreed to participate and successfully completed the study questionnaire. Most of the participants were female (67%) and held a bachelor’s degree in nursing (65.9%) (Table 1). Two-thirds of respondents (66%; *n* = 236) reported receiving information on hospital CDI protocols and policies, primarily during their job (62.3%; *n* = 147), at induction (18.6%; *n* = 44), or from an infection control nurse (14%; *n* = 33). The remaining respondents did not receive any information on these protocols.

In terms of basic microbiology and CDI-related knowledge (questions 1–4; Table 2), 30.5% (*n* = 109) of the respondents correctly identified *C. difficile* as an anaerobic bacillus, 42% (*n* = 151) correctly determined the percentage of normal healthy adults carrying toxin-producing *C. difficile* in their gut flora, and only 9% (*n* = 33) knew that toxic megacolon was caused by CDI. Most patients (58%; *n* = 209) were aware of the 2-day to 8-week incubation period for CDI. Regarding susceptibility risk factors (questions 5–8; Table 2), approximately 56% (*n* = 199) recognized predisposing factors for CDI, and 49% (*n* = 175) correctly identified cephalosporins, aminopenicillins, fluoroquinolones, and clindamycin as the most cited causes of CDI. Additionally, 24% (*n* = 86) of the respondents suggested limiting antibiotic use to effectively reduce symptomatic CDIs.

Regarding the detection and management of CDI (items 8–12; Table 2), approximately 19% (*n* = 67) of the respondents believed that not all *C. difficile*-associated diarrhea cases required treatment, whereas 81% (*n* = 291) agreed to treat all cases. Only 24% (*n* = 86) identified the cytotoxin B assay as the gold standard for identifying CDI. Interestingly, approximately 50% (*n* = 180) of the participants thought that stool culture was the gold standard. Few respondents 27% (*n* = 96) identified handwashing with soap and water as the most effective method for preventing the transmission of *C. difficile*. When asked about the first-line antibiotic for the treatment of *C. difficile*-associated diarrhea, 36% (*n* = 130) correctly selected oral metronidazole as the first-choice antibiotic, and 18% (*n* = 36) identified oral vancomycin as the second option for patients who failed to respond to oral metronidazole in cases of *C. difficile*-associated diarrhea.

Table 3 summarizes the association between the demographics of the respondents and their correct answers to different aspects of CDI. The results showed that female nurses have a significantly greater understanding of the risk factors associated with acquiring CDI (*p* < 0.001). Additionally, there was no significant association found between respondents’ age or educational qualifications and their understanding of other aspects of CDI.

## 4. Discussion

This study is the first to report on the knowledge and practices of nurses in Saudi Arabia regarding CDI diagnosis and management. The results of this survey revealed that, although many nurses had received information about CDI, only a few nurses could accurately identify its characteristics, risk factors, diagnosis, prevention, and treatment highlighting significant knowledge gaps. These findings indicate the need for targeted education and ongoing training to improve the awareness levels of CDI among nurses in this region.

Nurses play a key role in preventing CDI by employing infection control strategies, educating patients, and collaborating with the healthcare team [29]. Assessing their knowledge about CDI is essential for improving patient care and outcomes in healthcare settings. Therefore, this study evaluated the awareness of CDI among nurses in Saudi Arabia. Overall, one-third of nurses enrolled in our study reported not receiving education related to CDI. Similarly to our results, an Italian study found that 74% were aware of CDI procedures and guidelines, but only 46.5% implemented them [29]. This indicates a significant gap in both the awareness and adherence of CDI protocols among nurses, and the need for educational/training programs to be implemented in healthcare centers prior to their deployment.

Nurses in this study exhibited lower basic microbiological understanding, with only 30.4% correctly identifying *C. difficile* as an anaerobic bacillus, compared to higher rates in previous studies from the UK (54%) [31], Australia (77.8%) [29], and Italy (87%) [32]. Our study revealed a limited understanding of clinical conditions associated with CDI (9.2%) in contrast to 40% and 92% reported by Aroori et al. (2009) [32] and Comparcini et al. (2023), respectively [29]. Additionally, their degree of knowledge about incubation period of *C. difficile*-associated disease was also lower (58.4%) when compared to the nurses in the UK (70%) [31].

The risk factor-related awareness of our nurses was found to be better than the nurses in the UK [31]. For example, more than half of our study nurses recognized the risk factors linked to CDI compared to 37% of nurses in the UK. Approximately half of the nurses were aware of the antibiotics implicated in the development of CDI, while only 15% of UK nurses had this knowledge. Additionally, 24% recognized that reducing antibiotic use is the most effective way to manage CDI, compared to just 8% in the UK [31]. On the other hand, the awareness of nurses regarding key predisposing factors for CDI was consistent with the findings from an Italian study, where over half of the respondents demonstrated similar awareness [29]. These findings demonstrate notable differences in the knowledge and understanding among nurses, and the need for underlying factors driving the differences in the knowledge levels among them.

The analysis of nurse responses regarding the detection and management of CDI showed that only 18.7% of our nurses understood that not all cases of associated diarrhea require treatment, while a greater percentage recognized the cytotoxin assay as the gold standard for detection compared to UK nurses [31]. Only 26.8% of nurses correctly identified hand washing with soap and water as an effective method to prevent CDI transmission, which is within the range reported across other studies (28.5–38%) [29,31,32]. This misconception may stem from the belief that alcohol gel is sufficient, despite it not eliminating *C. difficile* spores like it does for MRSA [29,31,32]. In terms of treatment-related responses, over one-third of nurses (36.3%) identified oral metronidazole as the first-line treatment for CDI in comparison to 9–70% reported in previous studies [29,31]. Additionally, only 17.6% of nurses were aware that oral vancomycin is the second-line treatment for persistent CDI, compared to 27% reported previously [31]. Differences in the choice of first- and second-line drugs across studies can be attributed to variations in nurses’ training and education, updates to the clinical guidelines, regional practices, access to resources, and nurses’ experience with CDI cases.

Furthermore, analysis of the relationship between demographics and nurse responses to various aspects of CDI revealed that female nurses have a significantly better understanding of the risk factors for acquiring CDI. There was no notable correlation between age or educational qualifications and their awareness of other aspects of CDI. The findings of our study are partially in concordance with the study by Comparcini et al. (2023), which showed that female nurses and those with higher education levels showed significantly higher knowledge scores about CDI compared to male nurses and those with only nursing degrees. Additionally, nurses in long-term care wards had a higher mean knowledge score than those in medicine wards [29]. The varying awareness levels among nurses in the current study compared to previous research may be attributed to the differences in study design, population, ethnicity, and timing of the studies [29,30,31,32].

Overall, this study revealed significant knowledge gaps among Saudi Arabian nurses regarding CDI, which may have serious implications for clinical practice. These gaps can lead to inconsistent care practices, as nurses may employ varying methods due to the absence of standardized guidelines. This inconsistency can result in delayed diagnosis and treatment, exacerbating patient conditions and increasing the risk of CDI transmission. Additionally, inadequate infection control measures may arise from outdated or incomplete knowledge, increasing the potential for outbreaks. Limited access to the latest research and guidelines further hinders the application of evidence-based practices, challenging nurses in providing optimal care. To address these issues, it is important to promote continuous education and training for nursing staff, ensuring that they are equipped with current knowledge and skills. Supporting research initiatives and implementing robust infection control protocols are essential steps to enhance the quality of care and improve patient outcomes in clinical settings. Several tailored training programs, such as the Targeted Assessment for Prevention strategy and Harison College of Pharmacy program, are being implemented to improve nurses’ understanding and perceptions of CDI through specialized infection control training [33,34]. These initiatives stress the importance of antimicrobial stewardship, infection control practices, and multidisciplinary collaboration to effectively manage CDI [24,28].

Several caveats of this study merit consideration. This was a cross-sectional study; thus, no inferences could be made about causality. The sample size may not be representative of the national nursing population, and the demographic distribution (67% female) may affect the generalizability of the results. This study relied on self-reported data, which could have led to potential bias. Additionally, the validity and reliability of the questionnaire used in this study have not been established, warranting the cautious interpretation of the findings. This study used a descriptive methodological approach; therefore, inferential studies are required to confirm the study findings. Finally, this study highlighted the skills and procedures of Saudi nurses in managing CDI. However, it lacks a comparative analysis with nurses from other countries, which could provide a broader context for their knowledge and behavior relative to international standards.

## 5. Conclusions

In summary, this study revealed a significant lack of awareness among nurses in Saudi Arabia regarding various aspects of CDI, including its characteristics, risk factors, diagnosis, prevention, and treatment. This gap in knowledge among nurses could lead to increased infection rates, delayed diagnosis and treatment, higher healthcare costs and compromised patient safety. Additionally, it undermines the professional accountability of the nurses. Improving CDI education and training for nurses is crucial to address these knowledge gaps and enhance the quality of patient care.

## Figures and Tables

**Table 1:** Demographic characteristics of the nurses.

Characteristics	Nurses (N = 358)
Sex, *n* (%)	
Male	118 (33.0)
Female	240 (67.0)
Educational level, *n* (%)	
Diploma in nursing	42 (11.7)
Bachelor’s degree	236 (65.9)
Master’s degree	74 (20.7)
Doctor of Philosophy (PhD)	6 (1.7)

**Table 2:** Responses to the questions related to various aspects of CDI.

Item	Nurses (N = 358)
Section 1: Basic microbiology and illness caused by CDI, *n* (%)	
1.- What type of organism is *C. difficile*?	
Anaerobic bacillus Gram-positive coccus Gram-negative bacillus Fungus	109 (30.4) 181 (50.5) 41 (11.5) 27 (7.6)
2.- What percentage of normal healthy adults carry toxin-producing *C. difficile* in their gut flora?	
None 5% 15–70% 100%	63 (17.6) 151 (42.2) 132 (36.9) 12 (3.3)
3.- Which of the following conditions may be due to CDI?	
Pseudomembranous colitis Antibiotic-associated colitis Antibiotic-associated cases of diarrhea Toxic megacolon	96 (26.8) 94 (26.2) 135 (37.8) 33 (9.2)
4.- What is the incubation period of *C. difficile* associated disease?	
Less than 2 days 2 days to 8 weeks 8–10 weeks More than 10 weeks	65 (18.1) 209 (58.4) 59 (16.5) 25 (7.0)
Section 2: Risk factors that increase susceptibility to acquiring CDI, *n* (%)	
5.- Which of the following risk factors are associated with acquisition of *C. difficile*?	
Prolonged hospital stay Advanced age Immunosuppressant Usage of antacids and stool softeners All of the above	62 (17.3) 43 (12.0) 31 (8.7) 23 (6.4) 199 (55.6)
6.- Which of the following antibiotics are the most frequently cited causes of CDI?	
Cephalosporins Aminopenicillins Fluoroquinolones Clindamycin All of the above	69 (19.3) 38 (10.6) 37 (10.3) 39 (10.9) 175 (48.9)
7.- What is the single most successful measure in reducing symptomatic CDI?	
Antibiotic restriction Use of gloves while examining patients Washing hands Prophylactic use of metronidazole	86 (24.0) 55 (15.4) 175 (48.9) 42 (11.7)
Section 3: Detection and management of CDI, *n* (%)	
8.- Is it necessary to treat all cases of *C. difficile* associated diarrhea?	
Yes No	291 (81.3) 67 (18.7)
9.- What is the gold standard test for the identification of pathogenic CDI?	
Cytotoxin assay for detecting cytotoxin B ELISA Latex agglutination test Stool culture	86 (24.0) 58 (16.2) 34 (9.5) 180 (50.3)
10.- What is the most effective way of preventing the transmission of *C. difficile*?	
Hand washing with soap and water Rubbing hands with alcohol-based products Wearing gloves All of the above	96 (26.8) 35 (9.8) 20 (5.6) 207 (57.8)
11.- What is the first-line antibiotic choice for the treatment of *C. difficile* associated diarrhea?	
Oral vancomycin Oral metronidazole Intravenous vancomycin Intravenous metronidazole	89 (24.9) 130 (36.3) 70 (19.5) 69 (19.3)
12.- The treatment of choice in patients who failed to respond to oral metronidazole in *C. difficile* associated diarrhea is:	
Addition of oral vancomycin Switch over to oral vancomycin Switch over to intravenous metronidazole Addition of intravenous vancomycin Switch over to intravenous metronidazole and oral vancomycin	91 (25.4) 63 (17.6) 71 (19.8) 78 (21.8) 55 (15.4)

Abbreviations: CDI, *Clostridioides difficile* infection; ELISA, enzyme-linked immunosorbent assay. Note: Correct answers are underlined.

**Table 3:** Association between demographic characteristics of respondents and their correct answers to different aspects of CDI.

(a) Basic Microbiology and Illness Caused by CDI											
		What Type of Organism is *C. difficile*? (*n* = 109)		What Percentage of Normal Healthy Adults Carry Toxin-Producing *C. difficile* in Their Gut Flora? (*n* = 151)			Which of the Following Conditions May Be Due to CDI? (*n* = 33)			What Is the Incubation Period of *C. difficile*-Associated Disease? (*n* = 209)	*p*-Value
Age group, in years, *n* (%)											χ^2^ = 2.421; *p* = 0.877
≤25		7 (6.4)		15 (9.9)			3 (9.1)			22 (10.5)	
26 to 39		83 (76.1)		114 (75.5)			23 (69.7)			153 (73.2)	
≥40		19 (17.5)		22 (14.6)			7 (21.2)			34 (16.3)	
Sex, *n* (%)											χ^2^ = 0.453; *p* = 0.929
Female		74 (67.9)		106 (70.2)			22 (66.7)			148 (70.8)	
Male		35 (32.1)		45 (29.8)			11 (33.3)			61 (29.2)	
Educational level, *n* (%)											χ^2^ = 1.922; *p* = 0.927
Diploma in nursing		14 (12.8)		17 (11.3)			5 (15.2)			19 (9.1)	
Bachelor’s degree		67 (61.5)		95 (62.9)			19 (57.5)			136 (65.1)	
Master’s and PhD		28 (25.7%)		39 (25.8%)			9 (27.3%)			54 (25.8%)	
**(b) Risk Factors That Increase Susceptibility to Acquiring CDI**											
		**Which of the Following Risk Factors Are Associated with Acquisition of *C. difficile?*** **(*n* = 199)**			**Which of the Following Antibiotics are the Most Frequently Cited Causes of CDI?** **(*n* = 175)**				**What Is the Single Most Successful Measure in Reducing Symptomatic CDI?** **(*n* = 86)**		***p*-Value**
Age group, in years, *n* (%)											χ^2^ = 6.985; *p* = 0.137
≤25		25 (12.5)			15 (8.6)				17 (19.8)		
26 to 39		141 (70.9)			132 (75.4)				58 (67.4)		
≥40		33 (16.6)			28 (16.0)				11(12.8)		
Sex, *n* (%)											χ^2^ = 23.417; *p* < 0.001
Female		155 (77.9)			133 (76.0)				44 (51.2)		
Male		44 (22.1)			42 (24.0)				42 (48.8)		
Educational level, *n* (%)											χ^2^ = 6.409; *p* = 0.171
Diploma in nursing		18 (9.0)			17 (9.7)				8 (9.3)		
Bachelor’s degree		150 (75.4)			127 (72.6)				54 (62.8)		
Master’s and PhD		31 (15.6)			31 (17.7)				24 (27.9)		
**(c) Detection and Management of CDI**											
	**Is It Necessary to Treat all Cases of *C. difficile*-Associated Diarrhea?** **(*n* = 67)**		**What Is the Gold Standard Test for the Identification of Pathogenic CDI?** **(*n* = 86)**			**What Is the Most Effective Way of Preventing Transmission of *C. difficile*?** **(*n* = 96)**		**What Is the First-Line Antibiotic Choice for the Treatment of *C. difficile*-Associated Diarrhea?** **(*n* = 130)**		**The Treatment of Choice in Patients Who Failed to Respond to Oral Metronidazole in *C. difficile*-Associated Diarrhea Is** **(*n* = 63)**	***p*-Value**
Age group, in years, *n* (%)											χ^2^ = 8.904; *p* = 0.350
≤25	14 (20.9)		14 (16.3)			10 (10.4)		17 (13.1)		5 (7.9)	
26 to 39	47 (70.1)		57 (66.3)			68 (70.8)		95 (73.1)		47 (74.7)	
≥40	6 (9.0)		15 (17.4)			18 (18.8)		18 (13.8)		11 (17.4)	
Sex, *n* (%)											χ^2^ = 4.061; *p* = 0.398
Female	40 (59.7)		53 (61.6)			64 (66.7)		89 (68.5)		35 (55.6)	
Male	27 (40.3)		33 (38.4)			32 (33.3)		41 (31.5)		28 (44.4)	
Educational level, *n* (%)											χ^2^ = 3.657; *p* = 0.887
Diploma in nursing	6 (8.9)		14 (16.3)			11 (11.5)		13 (10.0)		8 (12.7)	
Bachelor’s degree	43 (64.2)		54 (62.8)			59 (61.5)		86 (66.2)		38 (60.3)	
Master’s and PhD	18 (26.9)		18 (20.9)			26 (27.0)		31 (23.8)		17 (27.0)	

Abbreviations: CDI, *Clostridioides difficile* infection; PhD, Doctor of Philosophy.

## Data Availability

The original contributions presented in this study are included in the article/Supplementary Materials. Further inquiries can be directed to the corresponding author(s).

## Supplementary Materials

The following supporting information can be downloaded at: https://www.mdpi.com/article/10.3390/nursrep15020074/s1, Supplementary File S1: *Clostridioides difficile* Infection Study Questionnaire.

## Public Involvement Statement

There was no public involvement in any aspect of this research

## Guidelines and Standards Statement

This manuscript was drafted against the Strengthening the Reporting of Observational Studies in Epidemiology (STROBE) checklist for cross-sectional research.

## References

[1] Al-Zahrani I.A. (2023). *Clostridioides* (*Clostridium*) *difficile*: A silent nosocomial pathogen. Saudi Med. J..

[2] Li C., Li Y., Huai Y., Liu S., Meng X., Duan J., Klena J.D., Rainey J.J., Wu A., Rao C.Y. (2018). Incidence and outbreak of healthcare-onset healthcare-associated *Clostridioides difficile* infections among intensive care patients in a large teaching hospital in China. Front. Microbiol..

[3] Lev V., Anbarchian T., Yao H., Bhat A., Britt P., Shieh L. (2024). Health care-associated *Clostridioides difficile* infection: Learning the perspectives of health care workers to build successful strategies. Am. J. Infect. Control.

[4] Alshannaq A.F., Kates A.E., Keating J.A., Mckinley L.L., Dixon J.W., Safdar N. (2025). Diverse sources and latent reservoirs of community-associated *Clostridioides difficile* infection. Clin. Infect. Dis..

[5] Turner N.A., Krishnan J., Nelson A., Polage C.R., Sinkowitz-Cochran R.L., Fike L., Kuhar D.T., Kutty P.K., Snyder R.L., Anderson D.J. (2024). CDC’s Hospital-onset *Clostridioides difficile* prevention framework in a regional hospital network. JAMA Netw. Open.

[6] Finn E., Andersson F.L., Madin-Warburton M. (2021). Burden of *Clostridioides difficile* infection (CDI)—A systematic review of the epidemiology of primary and recurrent CDI. BMC Infect. Dis..

[7] Marra A.R., Perencevich E.N., Nelson R.E., Samore M., Khader K., Chiang H.Y., Chorazy M.L., Herwaldt L.A., Diekema D.J., Kuxhausen M.F. (2020). Incidence and outcomes associated with *Clostridium difficile* infections: A systematic review and meta-analysis. JAMA Netw. Open.

[8] Alalawi M., Aljahdali S., Alharbi B., Fagih L., Fatani R., Aljuhani O. (2020). *Clostridium difficile* infection in an academic medical center in Saudi Arabia: Prevalence and risk factors. Ann. Saudi Med..

[9] Althaqafi A., Munshi A., Baghlaf B., Munshi E., Malakah M., Almarhabi H., Alharbi M., Alsaedi A. (2022). The prevalence, risk factors, and complications of *Clostridium difficile* infection in a tertiary care center, western region, Saudi Arabia. J. Infect. Public Health.

[10] Gurung B., Stricklin M., Wang S. (2024). Gut microbiota-gut metabolites and *Clostridioides difficile* infection: Approaching sustainable solutions for therapy. Metabolites.

[11] Rodriguez C., Taminiau B., Van Broeck J., Delmee M., Daube G. (2015). *Clostridium difficile* infection and intestinal microbiota interactions. Microb. Pathog..

[12] Mullish B.H., Allegretti J.R. (2021). The contribution of bile acid metabolism to the pathogenesis of *Clostridioides difficile* infection. Therap. Adv. Gastroenterol..

[13] Clarke L.M., Allegretti J.R. (2024). The epidemiology and management of *Clostridioides difficile* infection—A clinical update. Aliment. Pharmacol. Ther..

[14] Mullish B.H., Williams H.R. (2018). *Clostridium difficile* infection and antibiotic-associated diarrhoea. Clin. Med..

[15] Banawas S.S. (2018). *Clostridium difficile* infections: A global overview of drug sensitivity and resistance mechanisms. Biomed. Res. Int..

[16] Abad C.L.R., Safdar N. (2021). A review of *Clostridioides difficile* infection and antibiotic-associated diarrhea. Gastroenterol. Clin. N. Am..

[17] Eeuwijk J., Ferreira G., Yarzabal J.P., van Beest Holle M.R.-D.R. (2024). A systematic literature review on risk factors for and timing of *Clostridioides difficile* infection in the United States. Infect. Dis. Ther..

[18] Vakili B., Fateh A., Asadzadeh Aghdaei H., Sotoodehnejadnematalahi F., Siadat S.D. (2020). Intestinal microbiota in elderly inpatients with *Clostridioides difficile* infection. Infect. Drug Resist..

[19] Hosseini-Moghaddam S.M., Luo B., Bota S.E., Husain S., Silverman M.S., Daneman N., Brown K.A., Paterson J.M. (2021). Incidence and outcomes associated with *Clostridioides difficile* infection in solid organ transplant recipients. JAMA Netw. Open.

[20] Tariq R., Singh S., Gupta A., Pardi D.S., Khanna S. (2017). Association of gastric acid suppression with recurrent *Clostridium difficile* infection: A systematic review and meta-analysis. JAMA Intern. Med..

[21] Roghmann M.C., Andronescu L.R., Stucke E.M., Johnson J.K. (2017). *Clostridium difficile* colonization of nursing home residents. Infect. Control Hosp. Epidemiol..

[22] Furuya-Kanamori L., Marquess J., Yakob L., Riley T.V., Paterson D.L., Foster N.F., Huber C.A., Clements A.C. (2015). Asymptomatic *Clostridium difficile* colonization: Epidemiology and clinical implications. BMC Infect. Dis..

[23] Jump R.L., Donskey C.J. (2015). *Clostridium difficile* in the long-term care facility: Prevention and management. Curr. Geriatr. Rep..

[24] Kociolek L.K., Gerding D.N., Carrico R., Carling P., Donskey C.J., Dumyati G., Kuhar D.T., Loo V.G., Maragakis L.L., Pogorzelska-Maziarz M. (2023). Strategies to prevent *Clostridioides difficile* infections in acute-care hospitals: 2022 Update. Infect. Control Hosp. Epidemiol..

[25] Carter E.J., Greendyke W.G., Furuya E.Y., Srinivasan A., Shelley A.N., Bothra A., Saiman L., Larson E.L. (2018). Exploring the nurses’ role in antibiotic stewardship: A multisite qualitative study of nurses and infection preventionists. Am. J. Infect. Control.

[26] Ngam C., Hundt A.S., Haun N., Carayon P., Stevens L., Safdar N. (2017). Barriers and facilitators to *Clostridium difficile infection* prevention: A nursing perspective. Am. J. Infect. Control.

[27] Clarkin C., Quist S., Shamis R., King A.E., Shah B.M. (2019). Management of *Clostridioides difficile* infection. Crit. Care Nurse.

[28] Musuuza J.S., Hundt A.S., Carayon P., Christensen K., Ngam C., Haun N., Safdar N. (2019). Implementation of a *Clostridioides difficile* prevention bundle: Understanding common, unique, and conflicting work system barriers and facilitators for subprocess design. Infect. Control Hosp. Epidemiol..

[29] Comparcini D., Simonetti V., Segala F.V., Di Gennaro F., Bavaro D.F., Pompeo M.A., Saracino A., Cicolini G. (2023). Nurses’ Knowledge, attitudes and practices on the management of *Clostridioides difficile* infection: A cross-sectional study. Antibiotics.

[30] Almutairi M.S., Alnezary F.S., Alsuwaylim R.O., Alsulaymi I., Almohammed O.A., Thabit A.K. (2024). Assessment of knowledge and practice of healthcare providers in Saudi Arabia regarding *Clostridioides difficile* infection diagnosis and management: A cross-sectional questionnaire-based study. Infect. Drug. Resist..

[31] Aroori S., Blencowe N., Pye G., West R. (2009). *Clostridium difficile*: How much do hospital staff know about it?. Ann. R. Coll. Surg. Engl..

[32] Finnimore K., Smyth W., Carrucan J., Nagle C. (2023). Nurses’ knowledge, practices and perceptions regarding *Clostridioides difficile*: Survey results. Infect. Dis. Health.

[33] Clostridioides difficile Infection (CDI) Implementation Guide. https://www.cdc.gov/healthcare-associated-infections/media/pdfs/HAI-Toolkit-TAP-Strategy-CDI-Implementation-Guide-508.pdf.

[34] Treatment of Clostridioides difficile Infection. https://aub.ethosce.com/content/treatment-clostridioides-difficile-infection.

