# Supplementary material for: Knowledge Assessment of Hospital Nursing Staff in Saudi Arabia Regarding Clostridioides difficile Infection: A Descriptive Cross-Sectional Study
Source: Nurs Rep. 2025 Feb 19;15(2):74. doi: 10.3390/nursrep15020074 (PMC11858021; doi:10.3390/nursrep15020074)
Supplement: Supplementary file 1 [file nursrep-15-00074-s001.zip › nursrep-3339350-supplementary.pdf]

Statement of Consent: I have read the above information and have received answers to any questions I asked.

1. **What is your gender? (Mark only one option)**

   Answer: Male [ ] Female [ ]

2. **What is your age in years?**

   Answer: [ ] years

3. **What is your highest academic qualification? (Mark only one option)**

   Answer: Diploma [ ] Bachelors [ ] Masters [ ] PhD [ ]

4. **Have you received any information (either written, verbal or both) about trust policy regarding *C. difficile* infection?**

   Answer: Yes [ ] No [ ]

   If yes:

5. **What is your field of expertise?**

   Answer:

6. **When did you get the information?**

   [ ] At induction

   [ ] During your job

   [ ] Informal visit by the microbiologist

   [ ] Informal visit by the infection control nurse

7. **What type of organism is *C. difficile*?**

   [ ] Anaerobic bacillus

   [ ] Gram-positive coccus

   [ ] Gram-negative bacillus

   [ ] Fungus

8. **What percentage of normal healthy adults carry toxin-producing *C. difficile* in their gut flora?**

   [ ] None

   [ ] 5%

   [ ] 15–70%

   [ ] 100%

9. **Which of the following conditions may be due to *C. difficile* infection?**

   [ ] Pseudomembranous colitis

   [ ] Antibiotic-associated colitis

   [ ] Antibiotic-associated cases of diarrhea

[ ] Toxic megacolon

10. What is the incubation period of *C. difficile* associated disease?

[ ] Less than 2 days

[ ] 2 days to 8 weeks

[ ] 8–10 weeks

[ ] More than 10 weeks

11. Which of the following risk factors are associated with acquisition of *C. difficile*?

[ ] Prolonged hospital stay

[ ] Advanced age

[ ] Immunosuppressant

[ ] Usage of antacids and stool softeners

[ ] All of the above

12. Which of the following antibiotics are the most frequently cited causes of *C. difficile* infection?

[ ] Cephalosporins

[ ] Aminopenicillins

[ ] Fluoroquinolones

[ ] Clindamycin

[ ] All of the above

13. What is the single most successful measure in reducing symptomatic *C. difficile* infection?

[ ] Antibiotic restriction

[ ] Use of gloves while examining patients

[ ] Washing hands

[ ] Prophylactic use of metronidazole

14. Is it necessary to treat all cases of *C. difficile* associated diarrhea?

[ ] Yes

[ ] No

15. What is the gold standard test for the identification of pathogenic *C. difficile* infection?

[ ] Cytotoxin assay for detecting cytotoxin B

[ ] ELISA

[ ] Latex agglutination test

[ ] Stool culture

16. **What is the difference between the *C. difficile* responsible for recent outbreaks and the strain responsible for the outbreaks in 1980s and 1990s?**

    [ ] It produces greater amounts of cytotoxins A and B

    [ ] It carries an additional clostridial toxin

    [ ] It has increased sporulation capacity

    [ ] Fluoroquinolone resistance

    [ ] All of the above

17. **What is the most effective way of preventing transmission of *C. difficile*?**

    [ ] Hand wash with soap and water

    [ ] Rubbing hands with alcohol-based products

    [ ] Wearing gloves

    [ ] All of the above

18. **What is the first-line antibiotic choice for the treatment of *C. difficile* associated diarrhea?**

    [ ] Oral vancomycin

    [ ] Oral metronidazole

    [ ] Intravenous vancomycin

    [ ] Intravenous metronidazole

19. **The treatment of choice in patients who failed to respond to oral metronidazole in *C. difficile* associated diarrhea is:**

    [ ] Addition of oral vancomycin

    [ ] Switch over to oral vancomycin

    [ ] Switch over to intravenous metronidazole

    [ ] Addition of intravenous vancomycin

    [ ] Switch over to intravenous metronidazole and oral vancomycin
